# Clinical characteristics on admission predict in-hospital fatal outcome in patients aged ≥75 years with novel coronavirus disease (COVID-19): a retrospective cohort study

**DOI:** 10.1186/s12877-020-01921-0

**Published:** 2020-11-30

**Authors:** Zhihua Yu, Yuhe Ke, Jiang Xie, Hao Yu, Wei Zhu, Liqun He, Qiongli Zheng, Chuanwei Li, Jingya Lu, Songnan Li, Songnan Wen, Sheng Wei, Nian Liu, Li Wei, Rong Bai

**Affiliations:** 1 Department of Cardiology, Beijing Anzhen Hospital, Capital Medical University, No. 2 Anzhen Road, Chaoyang District, Beijing, 100029 China; 2 Department of Cardiology, First Hospital of Wuhan City, Wuhan, 430022 China; 3 Department of Respiratory and Critical Medicine, Beijing Anzhen Hospital, Capital Medical University, Beijing, 100029 China; 4 Department of Cardiology, Daping Hospital, Third Military Medical University, Chongqing, 400042 China; 5 Department of Epidemiology and Biostatistics, School of Public Health, Tongji Medical College, Huazhong University of Science and Technology, Wuhan, 430030 China; 6 Beijing Institute of Heart, Lung& Blood Vessel Diseases, Beijing, 100029 China; 7 National Clinical Research Center of Cardiovascular Diseases, Beijing, 100029 China

**Keywords:** Coronavirus disease, SARS-CoV-2, Elderly, Death, Prediction

## Abstract

**Background:**

Novel coronavirus disease 2019 (COVID-19) has become a worldwide pandemic and precise fatality data by age group is needed urgently. This study to delineate the clinical characteristics and outcome of COVID-19 patients aged ≥75 years and identify the risk factors of in-hospital death.

**Methods:**

A total of 141 consecutive patients aged ≥75 years who were admitted to the hospital between 12th and 19th February 2020. In-hospital death, clinical characteristics and laboratory findings on admission were obtained from medical records. The final follow-up observation was on the 31st March 2020.

**Results:**

The median age was 81 years (84 female, 59.6%). Thirty-eight (27%) patients were classified as severe or critical cases. 18 (12.8%) patients had died in hospital and the remaining 123 were discharged. Patients who died were more likely to present with fever (38.9% vs. 7.3%); low percutaneous oxygen saturation (SpO_2_) (55.6% vs. 7.3%); reduced lymphocytes (72.2% vs. 35.8%) and platelets (27.8% vs. 4.1%); and increased D-dimer (94.4% vs. 42.3%), creatinine (50.0% vs. 22.0%), lactic dehydrogenase (LDH) (77.8% vs. 30.1%), high sensitivity troponin I (hs-TnI) (72.2% vs. 14.6%), and N-terminal pro-brain natriuretic peptide (NT-proBNP) (72.2% vs. 6.5%; all *P* < 0.05) than patients who recovered. Male sex (odds ratio [OR] = 13.1, 95% confidence interval [CI] 1.1 to 160.1, *P* = 0.044), body temperature > 37.3 °C (OR = 80.5, 95% CI 4.6 to 1407.6, *P* = 0.003), SpO_2_ ≤ 90% (OR = 70.1, 95% CI 4.6 to 1060.4, *P* = 0.002), and NT-proBNP> 1800 ng/L (OR = 273.5, 95% CI 14.7 to 5104.8, *P* < 0.0001) were independent risk factors of in-hospital death.

**Conclusions:**

In-hospital fatality among elderly COVID-19 patients can be estimated by sex and on-admission measurements of body temperature, SpO_2_, and NT-proBNP.

**Supplementary Information:**

The online version contains supplementary material available at 10.1186/s12877-020-01921-0.

## Background

In December 2019, Wuhan city, the capital of Hubei province in China, became the centre of an outbreak of pneumonia caused by a novel coronavirus, which was designated severe acute respiratory syndrome coronavirus 2 (SARS-CoV-2) by the World Health Organization (WHO) [1–3]. The disease was then named coronavirus disease 2019 (COVID-19) [4] and was classified as a global pandemic in March 2020 [5].

The clinical presentation of COVID-19 varies from asymptomatic to mild flu-like symptoms to acute respiratory distress syndrome (ARDS). Other symptoms include neurological manifestations, such as confusion, impaired consciousness [6]; abnormalities associated with acute myocardial injury and heart failure [7]. These symptoms are more common in the elderly. As of September 2020, the overall mortality of COVID-19 was 3.2% globally and 5.2% in China [8]. However, mortality in the elderly (≥60 years) was consistently reported as high as 20% [9, 10] from different centers worldwide, suggesting that advanced age is associated with increased mortality and case fatality rate. Kremer S et al. [6] showed that old COVID-19 patients had more neurological symptoms. Neurological complications including stroke and encephalopathy have been seen in elderly COVID-19 populations which was associated with greater morbidity and increased social and economic burden. The study by Liu et al. [11] specifically compared the outcome of older COVID-19 patients with individuals at younger age and also found that mortality of elderly patients with COVID-19 was higher; however, their study included a very small sample size and did not identify any risk factor. Therefore, we aimed to investigate the clinical characteristics of COVID-19 patients aged ≥75 years and to identify risk factors that may predict in-hospital death.

## Methods

### Study design and participants

This was retrospective cohort study enrolling consecutive COVID-19 patients who were admitted to the First Hospital of Wuhan. This institution is one of the major tertiary teaching hospitals of Wuhan city and was selected as a COVID-19 designated hospital by the government on 11th February 2020. From 12th February 2020, by regulation the hospital only admitted confirmed COVID-19 patients who were in moderate, severe, or critical condition (defined below). Patients were 1) transferred from other non-designated hospitals, 2) transferred from the cabin hospitals because he/she progressed from a mild case to the stage of moderate or above and needed intensive care, or 3) directly admitted from the fever clinic of the First Hospital of Wuhan.

This study was approved by the Institutional Ethics Board of the First Hospital of Wuhan (No.202008) on February 12, 2020. Given the limited availability of human resource during the initial sweep of the pandemic, written consent was waived in the aforementioned ethic approval and thus oral consent was obtained from each patient to use his/her medical records for research purposes.

### Diagnosis criteria

All patients with COVID-19 enrolled in this study were diagnosed according to the WHO interim guidance [12] and the contemporary “Protocols for the Diagnosis and Treatment of 2019 New Coronavirus Pneumonia” issued by the National Health Commission of the People’s Republic of China [13]. In addition to clinical and lung imaging manifestations, all confirmed COVID-19 cases required at least one positive test result of the pathogen, i.e. the nucleic acid of the SARS-CoV-2 virus. Classification of mild, moderate, severe, or critical case was made using the following criteria [13]:

1. Mild: clinical symptoms are mild and no pneumonia manifestations can be found on lung imaging.

2. Moderate: symptomatic and typical ground-glass opacity lesions on lung imaging.

3. Severe: presenting with any of the following: respiratory rate of ≥30 breaths/min; oxygen saturation ≤ 93% at rest, arterial partial pressure of oxygen (PaO2)/oxygen concentration (FiO2) of ≤300 mmHg, > 50% lesion progression within 24-48 h.

4. Critical: meeting any of the following: respiratory failure requiring mechanical ventilation, presence of shock, extra-pulmonary organ failure.

### Treatment and discharge

In general, the management of COVID-19 patients followed the National’s protocol [13] and included antiviral treatment, anti-hypoxemia treatment, prevention of complications, and supportive care. Specific therapy was determined at the attending physician’s discretion. Approved by a multi-disciplinary specialist team, a COVID-19 patient could be discharged from hospital if he/she remained within the range of normal body temperature for at least 3 days, exhibited significant improvement in respiratory symptoms, had an SpO_2_ > 93% without oxygen inhalation, tested negative for SARS-CoV-2 nucleic acid twice consecutively (sampling> 24 h apart), exhibited significant improvement in lesions on lung computed tomography (CT), and had no other comorbidities or complications that required hospitalization.

### Clinical data collection and laboratory procedures

The electronic medical records of the patients were reviewed. Recorded information included demographic data, medical history, epidemiological history, underlying comorbidities, symptoms, signs, laboratory findings, radiological characteristics, and treatment. The date of disease onset was defined as the day when the symptom was first noticed. The last follow-up observation was on the 31st March 2020; by this date, all patients included in the present study were either deceased or had been discharged from hospital.

Laboratory confirmation of SARS-CoV-2 infection by real-time reverse-transcription polymerase chain reaction assay has been described elsewhere [14]. Briefly, throat swab samples were collected, and the total RNA was extracted within 2 h using the RNA Isolation Kit (Jiangsu Bioperfectus Technologies, Taizhou, China). Two target genes, including open reading frame lab (ORF lab) and nucleocapsid protein (N), were simultaneously amplified and tested.

Routine blood tests were conducted at the central laboratory of the hospital. These included a complete blood count, serum biochemical markers, electrolytes, myocardial enzymes, N-terminal pro-brain natriuretic peptide (NT-proBNP), D-dimer, C reaction protein (CRP), and procalcitonin (PCT). All patients underwent a chest CT scan immediately on admission to evaluate the presence of lung lesions and an electrocardiogram. Examinations were repeated as needed during hospitalization.

### Statistical analysis

Categorical variables were described as frequencies and percentages, and continuous variables were described using median and interquartile range (IQR) values. The primary endpoint of our study was the in-hospital outcome, according to which all the patients were divided into two groups: “Death” and “Discharged.” We used the Mann-Whitney U test, χ^2^ test, or Fisher’s exact test to compare the differences in variables between the two groups where appropriate. Univariate and multivariate non-conditional logistic regression models were used to explore the risk factors associated with in-hospital death. Clinical presentations and laboratory measurements that reported as continuous variable were categorised into binary data using the cut-off value of the normal range recommended by the central laboratory (Supplementary Table Reference range of laboratory values in the Additional file 1). All demographic, clinical, and laboratory data that exhibited significant or borderline significant differences both in continuous and categorical data comparison between the two groups were first tested in the univariate model. and then entered into the multivariate model if they achieved statistical significance (*P* < 0.1) in the univariate analysis. Logistic regression was used to develop a predictive model to the primary endpoint, of which the performance was justified by the area under the curve (AUC) of the ROC. The Z test was used to compare the performance of multiple models. All statistical analyses were performed using SPSS (version 24.0, SPSS Inc.) and MedCalc (version 19.1) software. A *P*-value of < 0.05 was considered statistically significant.

### Patient and public involvement reporting

This was a retrospective cohort study, and no patients were involved in the study design or in setting the research questions or the outcome measures directly. No patients were asked to advise on interpretation or writing up of results.

Study results will be summarized and released by professional social media and presented at relevant conferences after publication. The STONP prediction model will be shared for free by means of online tool and App.

## Results

### Patient demographic characteristics

Between 12th February and 19th February 2020, 1077 adult COVID-19 patients were admitted to the First Hospital of Wuhan; among them, 141 (13.1%) consecutive patients aged ≥75 years were included in the present study. The median age was 81 years (IQR,78–85), ranging from 75 to 97 years, and the majority were female (84, 59.6%). The median time from disease onset to hospitalization was 10 (IQR, 6.0–14.5) days.

Comorbidities were present in 76.6% of patients, with cardiovascular diseases including hypertension, coronary heart disease, and atrial fibrillation being the most common comorbidity (63.1%). Other comorbidities included endocrine disorders (25.5%, including diabetes, thyroid dysfunction), central nerve system disorders (21.3%, including stroke, Alzheimer’s disease, and Parkinson’s disease), respiratory diseases (15.6%, including chronic obstructive pulmonary disease, asthma and tuberculosis), chronic kidney disease (3.5%), and malignancy (5.7%). Epidemic investigation indicated that no patient had a history of direct contact with Huanan seafood market in Wuhan city.

### Patient outcome

Overall, 18 patients died (Death group) during hospitalization, corresponding to an in-hospital fatality rate of 12.8%. The cause of death was refractory shock in eight patients and acute respiratory distress syndrome (ARDS) in 10 patients (sustained hypoxia despite of ventilation in five patients). By the 31st March 2020, the remaining 123 patients had been discharged (Discharged group) from hospital after proper treatment. The median time from admission to discharge was 29 (IQR, 25.0–36.0) days and from illness onset to death or discharge was 39.0 (IQR, 31.0–48.0) days. The latter was significantly shorter in the Death group (26.5, IQR 14.8–38.8 days vs. 40.0, IQR 33.0–50.0 days in the Discharged group, *P* < 0.0001).

**Fig. 1:**
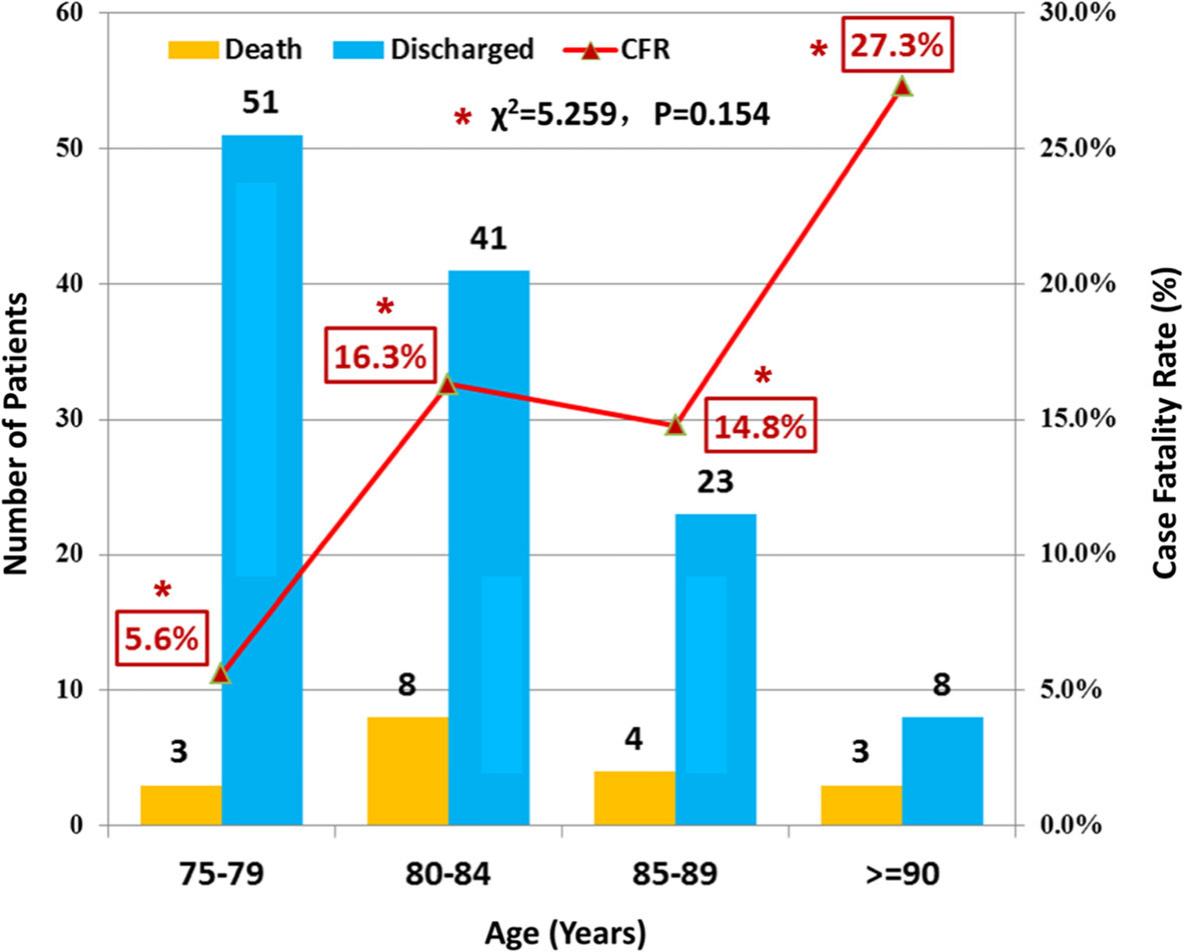
Number of patient dead or discharged and corresponding case fatality rate across age subgroups. Patients were stratified to 4 age subgroups by every 5-year increment. Bars and numerals on the top showed number of patients who were dead or discharged in each sub-group. Numeral in red frame indicated corresponding case fatality rate (CFR) in each sub-group. There was no significant different in CFR across 4 age subgroups (χ^2^ = 5.259, P = 0.154)

The age-stratified fatality rate is provided in Fig. 1 with the highest fatality rate at 27.3% in the ≥90 years subgroup. Although there seemed a trend of an increase of fatality rate by age, the comparison across all age subgroups did not achieve statistical significance (χ^2^ = 5.259, *P* = 0.154).

### Clinical presentations and laboratory tests on admission

The majority of patients (64.5%) reported being febrile since the onset of disease but only 11.3% of the population had fever (defined as an axillary temperature of > 37.3 °C) on arrival. Other common symptoms on admission were dry cough (53.9%), fatigue (39.0%), and expectoration (35.5%) as listed in Table 1. The incidence of all symptoms was comparable between the Death and the Discharged groups.

**Table 1:** Clinical Characteristics on Admission of Patients in the Discharged and Death Groups

	Total (***n*** = 141)	Discharged (***n*** = 123)	Death (***n*** = 18)	***Ρ*** value
**Median age(IQR)-years**	81.0(78.0–85.0)	80.0(77.0–85.0)	83.5(80.8–86.3)	**0.019**
**Sex-no.(%)**				0.056
Male	57(40.4)	46(37.4)	11(61.1)	
Female	84(59.6)	77(62.6)	7(38.9)	
**Severe / Critical case-no. (%)**	38(27.0)	24(19.5)	14(77.8)	**0.000**
**Days from illness onset to admission-median days(IQR)**	10.0(6.0–14.5)	10.0(6.0–15.0)	7.0(5.0–10.0)	0.069
**Comorbidity-no. (%)**				
Cardiovascular diseases	89(63.1)	76(61.8)	13(72.2)	0.391
Endocrine disorders	36(25.5)	30(24.4)	6(33.3)	0.601
Central nerve system disorders	30(21.3)	20(16.3)	10(55.6)	**0.000**
Respiratory diseases	22(15.6)	19(15.4)	3(16.7)	1.000
Malignancy	8(5.7)	7(5.7)	1(5.6)	1.000
Chronic kidney disease	5(3.5)	1(0.8)	4(22.2)	**0.001**
**Symptom, no. (%)**				
Fever	91(64.5)	79(64.2)	12(66.7)	0.840
Dry cough	76(53.9)	69(56.1)	7(38.9)	0.171
Fatigue	55(39.0)	50(40.7)	5(27.8)	0.296
Expectoration	50(35.5)	43(35.0)	7(38.9)	0.745
Dyspnea	46(32.6)	37(30.1)	9(50.0)	0.092
Anorexia	42(29.8)	37(30.1)	5(27.8)	0.842
Myalgia	12(8.5)	10(8.1)	2(11.1)	1.000
Nasal congestion	9(6.4)	9(7.3)	0(0)	0.503
Lethargy	9(6.4)	7(5.7)	2(11.1)	0.717
Pharyngalgia	6(4.3)	5(4.1)	1(5.6)	0.566
Diarrhea	6(4.3)	6(4.9)	0(0)	1.000
Dizziness	5(3.5)	3(2.4)	2(11.1)	0.122
Nausea	4(2.8)	4(3.3)	0(0)	1.000
Headache	4(2.8)	3(2.4)	1(5.6)	0.425
Vomiting	4(2.8)	4(3.3)	0(0)	1.000
**Sign**				
Temperature,median (IQR)-°C	36.5(36.3–36.8)	36.5(36.2–36.8)	36.8(36.5–37.9)	**0.003**
> 37.3-no. (%)	16(11.3)	9(7.3)	7(38.9)	**0.000**
HR,median (IQR)-bpm	84.0(76.0–93.5)	84.0(76.0–92.0)	86.5(75.0–96.5)	0.542
> 100-no. (%)	14(9.9)	13(10.6)	1(5.6)	0.808
RR, median (IQR)-bpm	20.0(19.0–22.0)	20.0(19.0–22.0)	22.0(19.8–25.0)	0.027
> 24-no. (%)	16(11.3)	11(8.9)	5(27.8)	0.051
SBP, median (IQR)-mmHg	133.0(123.5–146.5)	135.0(125.0–148.0)	126.5(119.3–139.0)	0.093
≤ 90-no. (%)	2(1.4)	0(0.0)	2(11.1)	0.016
S_P_O_2,_ median (IQR)-%	96.0(93.0–98.0)	97.0(95.0–98.0)	89.5(84.8–93.8)	**0.000**
≤ 90%-no. (%)	19(13.5)	9(7.3)	10(55.6)	**0.000**

*P* values denoted the comparison between Discharged and Death groups. Cardiovascular diseases included hypertension, coronary heart disease and atrial fibrillation; Endocrine disorders included diabetes mellitus and hypothyroidism; Central nerve system disorders included stroke, Alzheimer’s disease, and Parkinson’s disease; Respiratory diseases included chronic obstructive pulmonary disease, asthma and tuberculosis. *Bpm* beats per minute or breath per minute, *HR* heart rate, *RR* respiratory rate, *SBP* systolic blood pressure, *SpO*_*2*_ pulse oxygen saturation, *IQR* interquartile range

Some patients presented with signs suggesting unstable condition including heart rate of > 100 bpm (9.9%), respiratory rate of > 24 breaths/min (11.3%), systolic blood pressure of ≤90 mmHg (1.4%), and SpO_2_ ≤ 90% on room air (13.5%). When comparing between groups, there were significantly more patients in the Death group with these signs on admission (Table 1).

The laboratory test results on admission were summarized in Table 2. In the Death group, there was a markedly higher percentage of patients with abnormal findings in terms of the white blood cell count, lymphocyte count, neutrophil count, platelet count, CRP, PCT, D-dimer, blood urea nitrogen, creatinine, lactic dehydrogenase (LDH), N-terminal pro-brain natriuretic peptide (NT-proBNP), and hypersensitive troponin I (hs-TnI). All patients underwent a CT scan immediately before or after they were admitted to hospital; 37.6% had typical ground-glass opacity lesions in at least three lung lobes.

**Table 2:** Laboratory Findings on Admission in the Discharged and Death Groups

	Total (***n*** = 141)	Discharged (***n*** = 123)	Death (***n*** = 18)	***Ρ*** value
WBC- × 10^9^/L, median (IQR)	6.3(5.0–8.4)	6.2(5.0–8.1)	8.6(5.2–14.9)	0.064
> 10-no.(%)	18(12.8)	10(8.1)	8(44.4)	0.000
Lymphocyte- × 10^9^/L, median (IQR)	1.2(1.0–1.6)	1.3(1.0–1.6)	1.0(0.7–1.4)	**0.010**
≤1.1-no. (%)	57(40.4)	44(35.8)	13(72.2)	**0.003**
Neutrophil- × 10^9^/L, median (IQR)	4.0(3.0–6.2)	3.9(2.9–5.8)	7.2(3.1–13.9)	**0.022**
> 6.3-no. (%)	34(24.1)	24(19.5)	10(55.6)	**0.002**
Platelet- × 10^9^/L, median (IQR)	225.0(163.5–296.0)	234.0(172.0–301.0)	134(96.5–217.0)	**0.001**
≤100-no. (%)	10(7.1)	5(4.1)	5(27.8)	**0.002**
RBC- × 10^12^/L, median (IQR)	3.9(3.6–4.3)	3.9(3.6–4.3)	3.7(3.0–4.0)	0.023
≤4.0-no. (%)	84(59.6)	70(56.9)	14(77.8)	0.092
Hemoglobin- g/L, median (IQR)	120.0(109.5–130.0)	121.0(111.0–131.0)	112.0(100.0–119.0)	0.009
≤110-no. (%)	38(27.0)	30(24.4)	8(44.4)	0.132
CRP-mg/L, median (IQR)	13.2(3.2–39.1)	9.0(3.1–28.8)	106.0(21.9–150.0)	**0.000**
> 5.0-no. (%)	100(70.9)	82(66.7)	18(100.0)	**0.004**
Procalcitonin-ng/L, median (IQR)	40.0(20.0–100.0)	40.0(20.0–70.0)	360.0(97.5–1235.0)	**0.000**
> 500-no. (%)	9(6.4)	1(0.8)	8(44.4)	**0.000**
D-dimer-mg/L, median (IQR)	0.9(0.8–3.4)	0.9(0.8–2.7)	3.1(2.1–12.1)	**0.000**
> 1.0-no. (%)	69(48.9)	52(42.3)	17(94.4)	**0.000**
Creatinine-μmol/L, median (IQR)	69.0(58.0–98.0)	68.0(57.0–94.0)	96.0(64.3–133.3)	**0.026**
> 97-no. (%)	36(25.5)	27(22.0)	9(50.0)	**0.024**
BUN-mmol/L, median (IQR)	6.3(4.4–8.3)	5.4(4.1–7.5)	9.8(6.8–13.6)	**0.000**
> 7.1-no. (%)	47(33.3)	35(28.5)	12(66.7)	**0.001**
AST-IU/L, median (IQR)	25.0(20.0–33.5)	24.0(19.0–31.0)	33.0(23.8–55.5)	0.005
> 35-no. (%)	31(22.0)	24(19.5)	7(38.9)	0.121
ALT-IU/L, median (IQR)	19.0(13.0–26.5)	18.0(13.0–27.0)	22.0(13.0–27.3)	0.500
> 45-no. (%)	14(9.9)	14(11.4)	0(0.0)	0.277
LDH- IU/L, median (IQR)	218.0(168.5–327.5)	201.0(160.0–275.0)	485.5(257.3–811.3)	**0.000**
> 250-no. (%)	51(36.2)	37(30.1)	14(77.8)	**0.000**
TB-μmol/L, median (IQR)	11.5(9.1–15.7)	11.5(9.1–14.8)	12.5(9.0–18.0)	0.695
> 24-no. (%)	15(10.6)	12(9.8)	3(16.7)	0.632
Hs-TnI-ng/L, median (IQR)	14.0(8.0–25.0)	12.0(7.0–22.0)	41.0(24.8–168.0)	**0.000**
> 26-no. (%)	31(22.0)	18(14.6)	13(72.2)	**0.000**
CK-MB-IU/L, median (IQR)	7.0(5.0–11.0)	7.0(5.0–10.0)	8.5(4.8–13.0)	0.205
> 24-no. (%)	4(2.8)	2(1.6)	2(11.1)	0.079
CK-IU/L, median (IQR)	49.0(31.0–77.0)	44.0(30.0–70.0)	95.0(40.3–156.5)	0.010
> 170-no. (%)	11(7.8)	8(6.5)	3(16.7)	0.303
NT-proBNP-ng/L,median (IQR)	290.0(150.8–819.0)	260.0(124.0–512.0)	2362.5(1707.6–2978.3)	**0.000**
> 1800-no. (%)	21(14.9)	8(6.5)	13(72.2)	**0.000**
Ground-glass opacity lesions on CT				**0.006**
1–2 lung lobes, no. (%)	88(62.4)	82(66.7)	6(33.3)	.
≥ 3 lung lobes, no. (%)	53(37.6)	41(33.3)	12(66.7)	

*P* values denoted the comparison between Discharged and Death groups. *IQR* interquartile range, *WBC* white blood cell, *RBC* red blood cell, *CRP* C reaction protein, *BUN* blood urea nitrogen, *AST* aspartateaminotransferase, *ALT* alanineaminotransferase, *LDH* lactic dehydrogenase, *TB* total bilirubin, *Hs-TnI* hypersensitive troponin I, *CK-MB* creatinekinase-MB, *CK* creatine kinase, *NT-proBNP* N-terminal pro-brain natriuretic peptide, *CT* computed tomography

Overall, 38 patients (27%) were classified as severe or critical cases on admission and required immediate intensive care. These patients accounted for 77.8% of all cases of in-hospital death. This ratio was markedly higher than that in the Discharged group (24 severe/critical cases on admission of 123 discharged patients, 19.5%, *P* < 0.0001).

### Complications and treatment during hospitalization

After admission, 27.7% of the patients developed de novo arrhythmias including atrial fibrillation with rapid ventricular response, atrial flutter, atrial tachycardia, and first- or second-degree heart block, but no life-threatening arrhythmia was observed in our population. The incidence of arrhythmic occurrence in two groups was similar. Of the 123 patients who were eventually discharged, only seven (5.7%) deteriorated to ARDS during hospitalization, while in the Death group, this incidence was 55.6% (*P* < 0.0001). Deterioration to refractory shock was seen in eight patients who all died.

Arbidol was the most frequently (91.5%) used antiviral medicine. Most patients received antibiotics (76.6%), while 20.6% received glucocorticoid therapy. Mechanical ventilation, either non-invasive or invasive (IMV), was required in 21 cases (14.9%); six of these patients were intubated (IMV). Other treatments for COVID-19 are detailed in Table 3. Patients who received antibiotics, antifungal agents, or glucocorticoids; who needed immunoglobulin or blood transfusion; and who required mechanical ventilation were more likely to have a fatal outcome.

**Table 3:** Complication and Treatment during hospitalizationin the Discharged and Death Groups

	Total(***n*** = 141)	Discharged(***n*** = 123)	Death(***n*** = 18)	***Ρ*** value
**Complication-no.(%)**				
Arrhythmia	39(27.7)	32(26.0)	7(38.9)	0.391
ARDS	17(12.1)	7(5.7)	10(55.6)	**0.000**
Shock	8(5.7)	0(0)	8(44.4)	**0.000**
**Treatment-no.(%)**				
Antiviral therapy	131(92.9)	116(92.8)	15(93.8)	1.000
Oseltamivir	18(12.8)	17(13.8)	1(5.6)	0.546
Arbidol	129(91.5)	113(91.9)	16(88.9)	1.000
Oseltamivir+Arbidol	16(11.3)	15(12.2)	1(5.6)	0.666
Antibiotics	108(76.6)	90(73.2)	18(100.0)	**0.027**
Antifungal	6(4.3)	3(2.4)	3(16.7)	**0.028**
Glucocorticoid	29(20.6)	16(13.0)	13(72.2)	**0.000**
Immunoglobulin transfusion	15(10.6)	8(6.5)	7(38.9)	**0.000**
Blood transfusion	8(5.7)	5(4.1)	3(16.7)	0.107
**NIV-no.(%)**	15(10.6)	4(3.3)	11(61.1)	**0.000**
**IMV-no.(%)**	6(4.3)	1(0.8)	5(27.8)	**0.000**

*Ρ* value: comparison between Discharged and Death groups. *ARDS* acute respiratory distress syndrome, *NIV* noninvasive ventilation, *IMV* invasive mechanical ventilation

### Risk factors of in-hospital death

We tested the demographic and laboratory variables that exhibited a borderline significant or significant difference between the Discharged and Death groups in the univariate regression analysis, with the exception of age and CRP. Because there was collinearity between the variables of WBC, lymphocyte, and neutrophil, we excluded WBC and neutrophil. So totally 16 variables were included in the multivariate regression. The results indicated that male sex (odds ratio [OR] = 13.1, 95% confidence interval [CI]1.1 to 160.1, *P* = 0.044) and three on-admission check-ups including body temperature of > 37.3 °C (OR = 80.5, 95%CI 4.6 to 1407.6, *P* = 0.003), S_P_O_2_ of ≤90% without additional oxygen supply (OR = 70.1, 95%CI 4.6 to 1060.4, *P* = 0.002), and NT-proBNP of > 1800 ng/L (OR = 273.5, 95%CI 14.7 to 5104.8, *P* < 0.0001) were independent risk factors of fatal outcome (Table 4).

**Table 4:** Univariate and Multivariate Logistic Regression for Prediction of In-hospital Death

	Univariate OR (95%CI)	***Ρ*** value	Multivariate OR (95%CI)	***Ρ*** value
**Sex (Male)**	2.6(1.0–7.3)	0.062	13.1(1.1–160.1)	**0.044**
**Sign**				
Respiratory rate	3.9(1.2–13.0)	0.026		
Temperature-°C			80.5(4.6–1407.6)	**0.003**
≤ 37.3 °C	1(ref)			
> 37.3 °C	8.1(2.5–25.9)	0.000		
S_p_O_2_			70.1(4.6–1060.4)	**0.002**
> 90%	1(ref)			
≤ 90%	15.8(5.0–50.1)	0.000		
**Comorbidity (present vs. not present)**				
Central nerve system disorders	6.4(2.3–18.3)	0.000		
Chronic kidney disease	34.9(3.6–334.0)	0.002		
**Laboratory finding**				
WBC- × 10^9^/L				
≤ 10.0	1(ref)			
> 10.0	9.0(2.9–28.1)	0.000		
Lymphocyte count- × 10^9^/L				
> 1.1	1(ref)			
≤ 1.1	4.7(1.6–14.0)	0.006		
Neutrophil count- × 10^9^/L				
≤ 6.3	1(ref)			
6.3	5.2(1.8–14.5)	0.002		
Platelet count- × 10^9^/L				
> 100	1(ref)			
≤ 100	9.1(2.3–35.6)	0.002		
RBC- × 10^12^/L				
> 4	1(ref)			
≤ 4	2.7(0.8–8.5)	0.102		
Hemoglobin-g/L				
> 110	1(ref)			
≤ 110	2.5(0.9–6.9)	0.080		
PCT-ng/L				
≤ 500	1(ref)			
> 500	97.6(11.1–860.4)	0.000		
D-dimer-mg/L				
≤ 1.0	1(ref)			
> 1.0	23.2(3.0–180.0)	0.003		
Cr-μmol/L				
≤ 97	1(ref)			
> 97	3.6(1.3–9.8)	0.015		
BUN-mmol/L				
≤ 7.1	1(ref)			
> 7.1	5.0(1.8–14.4)	0.003		
LDH-IU/L				
≤ 250	1(ref)			
> 250	8.1(2.5–26.4)	0.000		
Hs-TnI-ng/L				
≤ 26	1(ref)			
> 26	15.2(4.8–47.7)	0.000		
NT-proBNP-ng/L			273.5(14.7–5104.8)	**0.000**
≤ 1800	1(ref)			
> 1800	37.4(10.6–131.2)	0.000		
Ground-glass opacity lesions on CT				
1–2 Lung lobes	1(ref)			
≥ 3 Lung lobes	4.0(1.4–11.4)	0.010		

*SpO*_*2*_ pulse oxygen saturation, *WBC* white blood cell, *RBC* red blood cell, *PCT p*rocalcitonin, *Cr creatinine*, *BUN* blood urea nitrogen, *LDH* lactic dehydrogenase, *Hs-TnI* hypersensitive troponin I, *NT-proBNP* N-terminal pro-brain natriuretic peptide, *CT* computed tomography, *CI* confidence interval

**Fig. 2:**
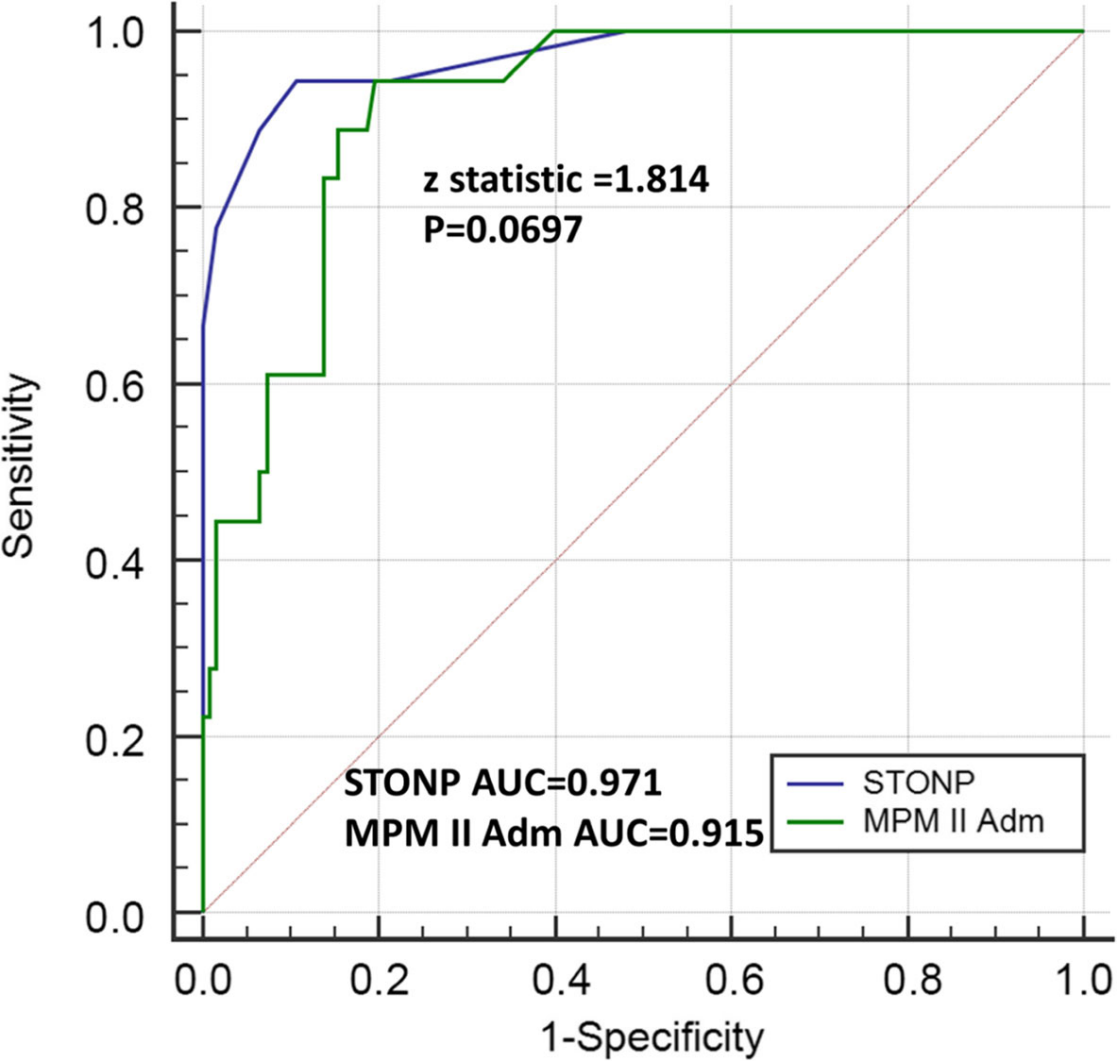
Receiver operator characteristic curve (ROC) of the STONP and the MPM II Adm model. Performance of the STONP and the MPM II Adm model was plotted in ROC and compared by the area under the curve (AUC). The predictive power of two models were comparable (z statistic = 1.814, *P* = 0.0697). STONP: *Sex,* *Temperature, SpO*_*2*_*,* and *NT-proBNP* model to predict in-hospital death of elderly COVID-19 patients; MPM II Adm: Mortality Probability Model II-Admission

To predict in-hospital death for elderly COVID-19 patients, a statistical model, namely *Sex,* *Temperature, S*_*P*_*O*_*2*_*,*and *NT-proBNP* (STONP), was developed using logistic regression and then the ROC curve was plotted. In the present derivation cohort, the AUC of the STONP model was 0.971 (95% CI 0.928 to 0.992) with a negative predictive value of 98.4% and a positive predictive value of 77.8%. As a validation cohort was unavailable at this time, we compared the STONP model with the Mortality Probability Models II-Admission (MPM-II Adm), which has been widely used in intensive care medicine to calculate the possibility of in-hospital death. The performance of the STONP model was comparable with that of the MPM-II Adm (AUC 0.915, 95% CI 0.856 to 0.955; z statistic 1.814, *P* = 0.0697 vs. STONP; Fig. 2). A web-based tool and an App (Android system only) of the STONP model are available at this hyperlink: https://janzhou.org/covid-19/stonp.html or via the barcodes provided in the Supplementary Figure.

## Discussion

Our study summarized the clinical characteristics and explored the risk factors of in-hospital death of elderly COVID-19 patients. We found that the overall fatality rate of COVID-19 patients aged ≥75 years was 12.8%; over > 25% of patients presented as severe or critical cases on admission and required intensive care; and patients who were male and presented with a body temperature > 37.3 °C, SpO_2_ ≤ 90%, and NT-proBNP> 1800 ng/L were at high risk of death. The STONP model including these four risk factors performed well in predicting in-hospital death.

Most of the published studies [6, 7, 11, 14] on COVID-19 were cross-sectional analysis with no follow-up. All patients in our study were admitted to hospital and reached an endpoint of either death or discharge. Further, unlike the data reported in studies during the early phase of the pandemic in Wuhan [10, 14] our data in the late phase when medial overwhelming had been resolved reflect the real-world outcome of elderly COVID-19 patients. Although it is well established that the risk of death from COVID-19 increases with older age [15, 16], the reported mortality rates in other studies were either retrieved from a general COVID-19 population or adjusted using statistical models [17, 18]. When the fatality rates are highly variable across age groups, it is critical to obtain data from age-specific cohort. Our study in patients ≥75 years served a timely response to the call by R. Bhopal [19] who emphasized the urgent need of precise data on COVID-19 by age group. We found that the overall fatality rate in elderly COVID-19 patients was 12.8%, but this rate did not significantly differ among septuagenarians, octogenarians, and nonagenarians. Our result is of unique importance to the aging countries.

Although elderly people might be most vulnerable to SARS-CoV-2 infection, elderly patients are not necessarily always severe or critical cases. On admission, 37.6% of our patients had lesions in at least three lung lobes, but the fatality rate (12.8%) was < 50% of this rate. It seems that the variable prognosis of COVID-19 patients was determined by the underlying health condition. Comorbidities were present in 76.6% of our patients. Previous studies [20] reported similar findings that non-survivors presented higher proportion of various co-existing chronic illness. The immunosenescence and malnutrition could lead to a deficiency in control of viral replication. The elderly are more prone to an uncontrolled activation of innate immune response that leads to cytokine release syndrome and tissue damage [21]. Nutritional deficits are most prevalent in older population, thus contributing to weakening of the immune system [22]. As indicated in our results, the median time from symptom onset to admission was shorter in deceased patients (7 days) than in patients who recovered (10 days), suggesting that rapid disease progression during the initial phase could be a sign of a poorer outcome. In contrast, the percentage of patients who were febrile (64.5%) or had a fever on admission (11.3%) was markedly lower than that reported in the general adult COVID-19 population (94 and 43.8%, respectively) [15, 23]. An elderly patient could be too weak to have the body temperature raised, which is actually a warning sign of serious condition. We also found that females accounted for the majority of cases in this elderly cohort, but male sex was a risk factor of in-hospital death. One possible explanation is that females generally have a longer life span than males, while Asian men have five-fold more ACE2-expressing cells [24], the target of the SARS-CoV-2 virus, in the lungs than Caucasians or females. However, if this was the case, male sex could lose its predictive power in patients of other ethnicities. Future studies should validate the STONP model in different ethnicities.

Thus far, there is no specific treatment for COVID-19 [25]. The empirical use of anti-viral agents does not seem to be associated with better outcome. Further, it is controversial to administer steroids in cases of viral pneumonia including COVID-19 [26–30]. In our cohort, glucocorticoid administration was not common (20% of cases), likely due to concerns of advanced age. Approximately 50% of these cases died accounting for > 70% of the deceased cases. Over 75% of patients were administered antibiotics, including all 18 deceased cases. Nevertheless, these results did not indicate that steroid or antibiotic use was a determinant of fatality in elderly COVID-19 patients.

One of the risk factors identified in our study was a body temperature of > 37.3 °C on admission. The fact that the median time from disease onset to hospitalization was 7 days in the Death group suggested that sustained fever was indicative of a poor outcome. We also identified NT-proBNP of > 1800 ng/L as a risk factor of death. This cut-off value had been adjusted for age and renal function. Although BNP is a sensitive marker of heart failure, it is a prognostic parameter in chronic lung disease and its secretion increases in pulmonary congestion of any reason [31], which is present after significant exudation in the lungs of COVID-19 patients [32]. Meanwhile, a higher NT-proBNP level could be a result of myocardial injury, which was observed in > 20% of our patients.

We believe that the four independent risk factors of in-hospital death identified in our elderly COVID-19 population reflected the patients’ vulnerability to the virus (male sex), progression of the disease (temperature on admission), severity of lung lesions (S_P_O_2_ on room air, NT-proBNP), and function of other organs (NT-proBNP). Based on this finding, we developed the STONP model with the aim of rapidly detecting patients at high risk of death upon admission with minimum measurements. The performance of the STONP model was comparable to that of the MPM-II Adm which was designed to use a series of signs and measurements on admission to predict the probability of death [33]. This model, consisting of 15 parameters, has been validated in > 10,000 cases [34] including those with similar conditions as COVID-19. It seems that our STONP model specifically established for elderly COVID-19 patients is simpler and easier to use than the MPM-II Adm. In addition, there are other models have been established to predict death or organ failure in patients in intensive condition including the quick Sequential Organ Failure Assessment (qSOFA) score [35]. The qSOFA allows identifying patients with suspected infection who are at greater risk for a poor outcome outside the intensive care unit (ICU) which theoretically applies to COVID-19 patients. In comparison with MPM-II Adm and our STONP model, the qSOFA scale uses 3 clinical variables without any laboratory parameters. It is known for convenient, bedside use but the selected clinical variables (low blood pressure SBP ≤ 100 mmHg, high respiratory rate ≥ 22 breaths per min, altered mentation Glasgow coma scale< 15) were too general to reflect the features of any specific disease. Although not directly compared in our study, one could expect a very low sensitivity of the qSOFA performance in COVID-19 population.

Our study has some notable limitations. First, this study reflected experience from single centre, single counry with a relatively small sample size. Second, it specifically addressed the characteristics of elderly COVID-19 patients; therefore, the results may not apply to younger patients. Third, we did not acquire data on patients’ status after discharge. Finally, the STONP model was not validated in an external cohort. Investigators from around the world may use the free access to the STONP model and validate it in different population.

## Conclusions

In this age-stratified cohort, we found that the overall case fatality rate of COVID-19 patients aged ≥75 was 12.8% and this rate was similar among septuagenarians, octogenarians, and nonagenarians. The STONP model, consisting of sex and simple on-admission measurements including body temperature, SpO_2_, and NT-proBNP, can aid the early detection of elderly COVID-19 patients at high risk of in-hospital death.

## Supplementary Information

**Additional file 1: Supplementary Table** Reference range of laboratory values and STONP model. **Supplementary Figure** Barcode links to STONP online tool and App.

**Additional file 2: Table 1.** Results of qSOFA score statistical analysis. **Figure 1** Mann Whitney U Test of qSOFA in the Discharged and Death Groups. **Figure 2** ROC Curve of qSOFA Score.

## Data Availability

Data are stored by First Hospital of Wuhan City on servers with security according to the rules given by Chinese law. The data can be made available by reasonable request to the corresponding author.

## Abbreviations

ARDS: Acute respiratory distress syndrome
ACE2: Angiotensin-converting enzyme 2
ALT: Alanineaminotransferase
AST: Aspartateaminotransferase
BUN: Blood urea nitrogen
CK-MB: Creatine kinase-MB
COVID-19: Coronavirus Disease 2019
CK: Creatine kinase
CT: Computed tomography
CRP: C-reactive protein
Hs-TnI: Hypersensitive troponin I
LDH: Lactic dehydrogenase
NT-proBNP: N-terminal pro-brain natriuretic peptide
RBC: Red blood cell
RNA: Ribonucleic acid
SARS-CoV-2: Severe acute respiratory syndrome coronavirus 2
SBP: Systolic blood pressur
SpO2: Arterial partial pressure of oxygen
TB: Total bilirubin
WBC: White blood cell
