# Supplementary material for: Clinical characteristics on admission predict in-hospital fatal outcome in patients aged ≥75 years with novel coronavirus disease (COVID-19): a retrospective cohort study
Source: BMC Geriatr. 2020 Nov 30;20:514. doi: 10.1186/s12877-020-01921-0 (PMC7702221; doi:10.1186/s12877-020-01921-0)
Supplement: Supplementary file 1 — Additional file 1: Supplementary Table Reference range of laboratory values and STONP model. Supplementary Figure Barcode links to STONP online tool and App. [file 12877_2020_1921_MOESM1_ESM.pdf]

Supplementary Table Reference range of laboratory values

**Laboratory indicators**	**Reference Range**
White blood cell count-$\times 10^9$/L	3.5～9.5
Neutrophil count - $\times 10^9$/L	1.8～6.3
Lymphocyte count - $\times 10^9$/L	1.10～3.20
Red blood cell count-$\times 10^{12}$/L	3.80～5.10
Haemoglobin– g/L	115～150
Platelet count - $\times 10^9$/L	125～350
C-reactive protein – mg/L	0.00～5.00
PCT-ng/L	0～500
D-dimer-mg/L	0.00～1.00
ALT-IU/L	7～45
AST-IU/L	13～35
TB-µmol/L	2.0～24.0
BUN-mmol/L	2.5～7.1
Cr-µmol/L	44～97
LDH- IU/ L	114～250
CK- IU/L	0～170
CK-MB- IU/L	0.0～24.0
Hs-TnI- ng/L	0～26
NT-proBNP-ng/L	<450(<50yrs)<br><900(50～75yrs)<br><1800(>75yrs)

Supplementary Figure

1. Barcode links to STONP online tool.

2. Barcode links to STONP App (Android system only).
