# Supplementary material for: Clinical characteristics on admission predict in-hospital fatal outcome in patients aged ≥75 years with novel coronavirus disease (COVID-19): a retrospective cohort study
Source: BMC Geriatr. 2020 Nov 30;20:514. doi: 10.1186/s12877-020-01921-0 (PMC7702221; doi:10.1186/s12877-020-01921-0)
Supplement: Supplementary file 2 — Additional file 2: Table 1. Results of qSOFA score statistical analysis. Figure 1 Mann Whitney U Test of qSOFA in the Discharged and Death Groups. Figure 2 ROC Curve of qSOFA Score. [file 12877_2020_1921_MOESM2_ESM.pdf]

# the Supplementary File for reviewer#1

**Table 1qSOFA score in the Discharged andDeath Groups**

| qSOFA score | Total(n=141) | Discharged(n=123) | Death(n=18) |
|---|---|---|---|
| 0 | 92(65.2) | 88(71.5) | 4(22.2) |
| 1 | 39(27.7) | 32(26.0) | 7(38.9) |
| 2 | 9(6.4) | 3(2.4) | 6(33.3) |
| 3 | 1(0.7) | 0(0.0) | 1(5.6) |

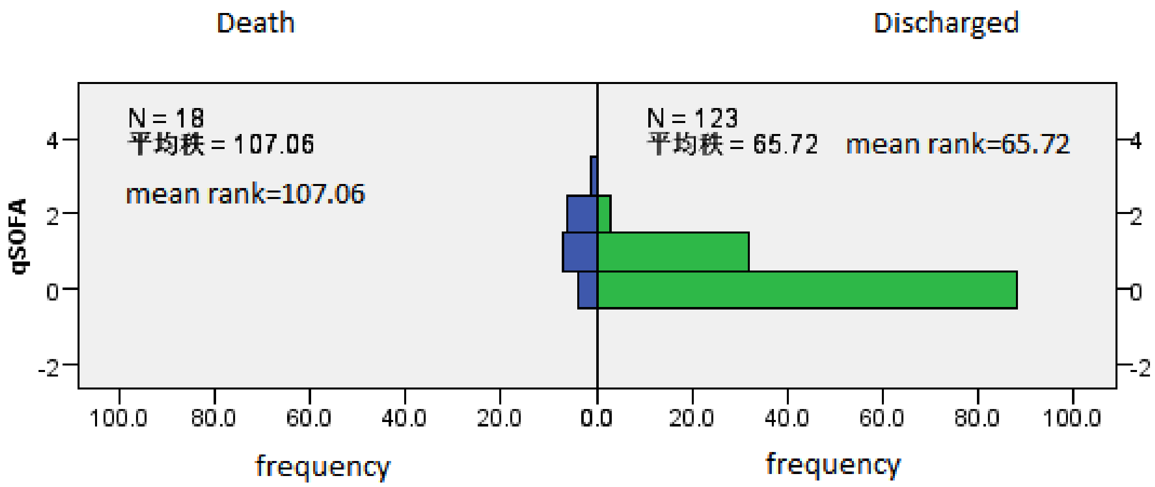


**Figure 1 Mann Whitney U Test of qSOFA in the Discharged and Death Groups**

| | |
|---|---|
| Total N | 141 |
| Mann Whitney U | 458.000 |
| Wilcoxon W | 8,084.000 |
| Test statistics | 458.000 |
| Standard error | 135.503 |
| Progressive significance（two sides） | .000 |

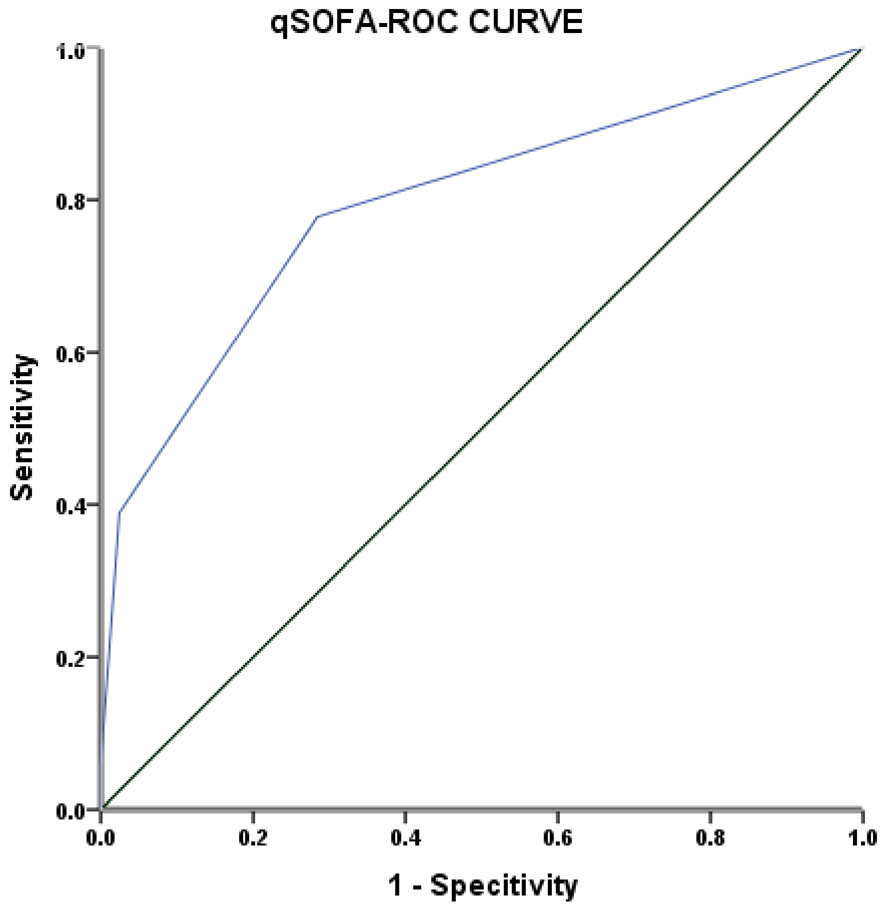


**Figure 2 ROC Curve of qSOFA Score**

| AUC | Standard error | Progressive significance | 95%CI |
|---|---|---|---|
| 0.793 | 0.065 | 0.000 | （0.667-0.920） |

| qSOFAScore ≥ | Sensitivity | 1-Specitivity |
|---|---|---|
| 0.50 | 0.778 | 0.285 |
| 1.50 | 0.389 | 0.024 |
| 2.50 | 0.056 | 0.000 |
| 4.00 | 0.000 | 0.000 |
